# Articular cartilage thickness alterations in hind limb of young and aged PAC1 gene-deficient mice

**DOI:** 10.1007/s00441-026-04066-5

**Published:** 2026-04-20

**Authors:** Csaba Fillér, Lili Sarolta Kovács, Kálmán Rácz, Yonatan Segal, Judit Vágó, Anna Tóth, Vince Szegeczki, Adél Jüngling, Péter Gergely, Róza Zákány, Dóra Reglődi, Tamás Juhász

**Affiliations:** 1 Department of Anatomy, Histology and Embryology, Faculty of Medicine, University of Debrecen, Nagyerdei Krt. 98, 4032 Debrecen, Hungary; 2 Department of Forensic Medicine, Faculty of Medicine, University of Debrecen, Nagyerdei Krt. 98, 4032 Debrecen, Hungary; 3 Department of Anatomy, Medical School, HUN-REN-PTE PACAP Research Team, University of Pécs, Szigeti Út 12, 7624 Pécs, Hungary

**Keywords:** Cartilage thickness, PAC1 receptor, Joints of hind limb, Aging, Sox9

## Abstract

Pituitary adenylate cyclase-activating polypeptide (PACAP) is a neuropeptide which was shown to be released in the hypothalamo-hypophyseal system but subsequently demonstrated in the entire nervous system and nearly all peripheral organs, including skeletal elements. PACAP has an important function in the regulation of chondrogenic differentiation, protecting in vitro chondrogenesis during various stresses and in osteogenesis. PACAP knockout (KO) mice show early signs of aging. Its most potent receptor is PAC1-R, the activation of which leads to enhanced Sox9 expression and subsequently, increase in the expression of collagen type II, glycosaminoglycans, and aggrecan. In the present experiments, we investigated the effect of the absence of PAC1 receptor in PAC1 KO homozygous and heterozygous mice focusing on joints of hind limb in young and aged animals. Thickness and extracellular matrix content of articular cartilage of joints increased in the absence of PAC1 receptor with aging. A thicker cartilage was detected in aged animals in mechanically affected joints. Interestingly, the disturbance of PACAP signaling pathways increased the nuclear translocation of P-Sox9 transcription factor in various joints. In summary, the alteration of PAC1 receptor regulated signalization elevated cartilage formation and protected cartilage architecture during aging suggesting a balancing effect of the receptor in chondrogenesis.

**Supplementary Information:**

The online version contains supplementary material available at 10.1007/s00441-026-04066-5.

## Introduction

Articular cartilage is a specialized type of connective tissue found in the joints of the skeletal system. Its primary function is to provide a smooth, low-friction surface for joint movement, as well as to distribute mechanical loads and absorb shock during weight-bearing activities. The unique structure of articular cartilage is crucial for its mechanical properties and overall function within the joint space. The injuries of the articular surface diminish the weight-bearing capacity of the joints (Carballo et al. 2017). As this tissue is avascular and aneural, its regeneration ability is very low (Redondo et al. 2018). Nutrients can reach the surface via synovial fluid, and in the lack of innervation and blood vessels, a very low amount of hormones can reach the articular surface (Wang et al. 2013). The structure of articular cartilage can be divided into different zones, each with distinct cellular and extracellular components. The superficial zone is the outermost layer of cartilage and contains a high density of collagen fibers oriented parallel to the joint surface. This zone is responsible for resisting shear forces during joint movement (Eschweiler et al. 2021). The intermediate or transitional zone is characterized by larger, randomly oriented collagen fibers running perpendicular to the articular facet and abundant proteoglycan (PG) content, which provides compressive strength to the cartilage (Eschweiler et al. 2021). The deep zone is the innermost layer of cartilage and contains the highest concentration of PGs, helping the absorption and distribution of mechanical loads within the joint. Finally, the calcified zone is the transition zone between the articular cartilage and the underlying subchondral bone, where mineralization of the cartilage matrix occurs (Cohen et al. 1998).

The structure of articular cartilage is composed primarily of water, collagen fibers, and proteoglycans. The collagen fibers are arranged in a parallel orientation within the cartilage matrix close to the surface and perpendicularly in the intermediate and deep zone, providing tensile strength and flexibility to the tissue. These fibers are primarily made up of collagen type II, which is essential for the structural integrity of the cartilage. However, around the chondrons, collagen type VI is the most abundant form, located circularly, forming a unique structure (Gilbert et al. 2021). In addition to collagen, the PGs within the cartilage matrix play a crucial role in maintaining the tissue’s hydration and mechanical properties as they have high negatively charged glucosaminoglycans (GAG) binding to them, such as chondroitin sulfate and keratan sulfate. Proteoglycans are large molecules composed of a protein core and GAG side chains, which attract and retain water within the cartilage matrix, such as aggrecan. This water content is essential for lubricating the joint surface and providing cushioning during joint movement. The unique structure of articular cartilage allows it to withstand repetitive loading and maintain joint function throughout life (Henao-Murillo et al. 2021). The chondrocytes are postmitotic cells with an extremely long lifespan. However, this structure is also vulnerable to damage and degeneration, particularly with aging or traumatic injury. Osteoarthritis is a common degenerative joint disease characterized by the breakdown of articular cartilage, leading to pain, inflammation, and loss of joint function (Abramoff and Caldera 2020). Understanding the molecular structure of articular cartilage is crucial for developing effective treatments and interventions to preserve joint health and function.

Pituitary adenylate cyclase-activating polypeptide (PACAP) is a neuropeptide that has been implicated in various physiological processes, including neurotransmission (Pinhasov et al. 2011), neuroprotection (Reglodi et al. 2018) and inflammation regulation (Vaudry et al. 2009). In recent years, studies have indicated that PACAP may also play a role in the regulation of chondrogenesis (Juhasz et al. 2014a, 2015a) and osteogenesis (Juhasz et al. 2014b). Several research studies have investigated the effects of PACAP on cartilage metabolism, with promising results. It has been found that PACAP could stimulate the synthesis of cartilage extracellular matrix components, such as collagen and proteoglycans in chondrocytes (Juhasz et al. 2014a, 2015b). This indicates that PACAP may have an anabolic effect on cartilage, promoting the production of essential structural components that are necessary for cartilage integrity. In addition to its effects on cartilage matrix synthesis, PACAP has also been shown to have anti-inflammatory properties (Toth et al. 2020) that may be beneficial for cartilage health. It also decreases the activity of matrix degrading enzymes such as MMP1 and ADAMTS4 (Szentleleky et al. 2019) or prevents the harmful effects of increased mechanical stress (Juhasz et al. 2015b; Szentleleky et al. 2019) and has a protective effect in oxidative stress (Juhasz et al. 2014a) in vitro. This suggests that PACAP may help to maintain chondrocyte viability and prevent cell death, which is essential for the maintenance of healthy cartilage.

PACAP exerts its effects via VPAC1, VPAC2 and PAC1 receptors with different affinities. The PAC1-R is a member of the vasoactive intestinal polypeptide (VIP) receptor family (Hirabayashi et al. 2018). It is a G protein-coupled receptor that plays a crucial role in various physiological processes, including neuroprotection, neuronal development, and the regulation of the endocrine and immune systems (Shen et al. 2013). The PAC1-R is a seven-transmembrane domain receptor that is coupled to G proteins, specifically Gs and Gq proteins. It is encoded by the ADCYAP1R1 gene located on chromosome 7p14.3 and its ligand-binding domain is located in the N-terminal extracellular region, which interacts with PACAP and VIP with high affinities (Kobayashi et al. 2020). Upon ligand binding, the receptor undergoes conformational changes that result in the activation of downstream signaling pathways (Wang et al. 2020). The intracellular domain of the receptor interacts with G proteins, which initiate the activation of adenylate cyclase (AC) and subsequently activate PKA. Activation of the latter kinase leads to the increased phosphorylation of transcription factors such as CREB and Sox9 (Juhasz et al. 2014a). The nuclear translocation of these transcription factors can increase extracellular matrix production of cartilage.

In conclusion, the PAC1-R is a critical signaling molecule that regulates a wide range of physiological processes in the body. Its role in neuroprotection, neuronal development, endocrine regulation and immune modulation makes it an attractive target for therapeutic interventions (Arimura and Shioda 1995; Delgado et al. 2003; Rivnyak et al. 2018). On the other hand, its loss of function is not clarified in cartilage formation, or its pivotal role is also unclear although its expression has been demonstrated in cartilage. In this study we followed the structure of articular cartilage of knee, intertarsal (IT), tarsometatarsal (TMT), metatarsophalangeal (MTP), and interphalangeal (IP) joints of young and aged wild type (WT), homozygous, and heterozygous PAC1 knockout (KO) mice.

## Material and methods

### Animals

Generation and maintenance of the PAC1-deficient mice has been established by Hashimoto et al. (Hashimoto et al. 2000). Genotype was tested with PCR reactions. For the experiments we sacrificed newborn (“young”) and 1-year-old (“aged”) wild type (WT, *n* = 10–10) and homozygous PAC1-deficient (PAC1 KO, *n* = 5) and heterozygous PAC1 deficient mice (PAC1 HZ, *n* = 10–10). PAC1 homozygous gene deficient mice showed a high mortality after 2 weeks of birth while the PAC1 heterozygous littermates stayed alive and aged similarly to WT littermates. Animals were fed and watered ad libitum, under light/dark cycles of 12/12 h. Hind limbs were removed after sacrificing the mice with an overdose of pentobarbital sodium (100 mg/kg bw). All procedures were performed in accordance with the ethical guidelines approved by the University of Pécs (permission number: BA02/2000–15024/2011). Hind limbs were further dissected and separated at the level of the ankle to foot and knee joints specimens.

### Histological analysis

Samples were washed in PBS (phosphate buffer solution) three times and fixed in 10% formalin fixative for 72 h. Bones were decalcified in 4% EDTA (Sigma-Aldrich, MO, USA) for four weeks until the bones and cartilage tissue became soft. Decalcifying solution was washed out with PBS for 30 min, and samples were embedded in paraffin. Serial sections of 7 µm thick slides were done with microtome (Leica, Wetzlar, Germany). The samples were stained with dimethyl-methylene blue (DMMB) dissolved in water (Sigma-Aldrich, MO, USA) and picrosirius red staining (Sigma-Aldrich, MO, USA) according to the instructions of the manufacturer. Slides were covered with DPX (Sigma-Aldrich, MO, USA). Histological slides stained with DMMB were examined with a light microscope BX53 Olympus (Olympus, Tokyo, Japan) with constant camera and exposure settings. Separate photos were made of the knee, IT, TMT, MTP, and IP joints.

### Measurements in polarization light microscopy

The samples stained with picrosirius red were examined with polarization lens where the polarized light plane was turned with λ/4 and analyzed by a λ/4 compensator with constant camera and exposure settings in BX53 Olympus microscope (Olympus, Tokyo, Japan). Photos were taken in normal light and then in polarized light with constant camera settings. Separate photos were done by knee, IT, TMT, MTP, and IP joints. A 1 mm wide region of articular cartilage was analyzed. Semiquantitative polarization light microscopy (PLM) scoring system was used on knee joints where scores range between 0 and 5, with lower score indicating degrading cartilage; a score of 0 describes cartilage specimens that have sparse patches of birefringence that are neither parallel nor perpendicularly orientated indicating disorganized cartilage (Mantripragada et al. 2021). Furthermore, the pixel intensity of green (thin) and red (thick) collagen fibers were also measured by ImageJ 1.40 g freeware in all joints and semiquantitative data were given and normalized to the data of young and/or aged WT animals.

### Measurement of thickness measurement of articular cartilage

For the measurement of articular cartilage thickness, a customized mathematical formula was used as it was described by Szegeczki et al. (Szegeczki et al. 2019) on DMMB stained slides, under 10 times magnification objective at least in 5 independent samples in all groups of young and aged animals. Five individual measurements were performed on each joint of interest.

### Immunohistochemistry

The localization of P-Sox9 was visualized with immunohistochemistry by using a polyclonal antibody (Sigma-Aldrich MO, USA, P-Sox9). Following deparaffinization in descending alcohol raw, samples were incubated 3 × 10 min in PBS (phosphate-buffered saline) and bovine serum albumin (BSA) (Amresco, CA, USA) was used to block the unspecific binding sites at 37℃ for 30 min; then slides were washed 3 × 10 min in PBST. The primary antibody of P-Sox9 at a 1:800 dilution was used overnight at 4℃. PBS was used to wash out the unbound primary antibody, then the secondary antibody, anti-rabbit-Alexa555 in a dilution of 1:1000 (Invitrogen, MA, USA) was applied. Slides were covered with DAPI (Vector Laboratories, CA, USA) to visualize the nucleus. Fluorescent images were taken with an Olympus FV1000S confocal microscope (Olympus Co., Tokyo, Japan) using a × 60 oil immersion objective (NA: 1.3) with the application of a 543 nm laser beam. The average pixel time was 4 μs. Z-stack image series of 1 μm optical thickness were recorded in sequential scan mode with constant settings; photos were taken passing through the nuclear plane. Images of Alexa555 and DAPI were overlaid using Adobe Photoshop version 10.0 software.

### Statistical analysis

All data are representative of at least five independent experiments. Where applicable, data are expressed as mean ± SEM. Statistical analysis was performed by Student’s *t*-test. Threshold for statistically significant differences as compared to respective control (wild-type animals) was set at *p < 0.05.

## Results

### Altered morphology in PAC1 gene-deficient knee joints

**Fig. 1:**
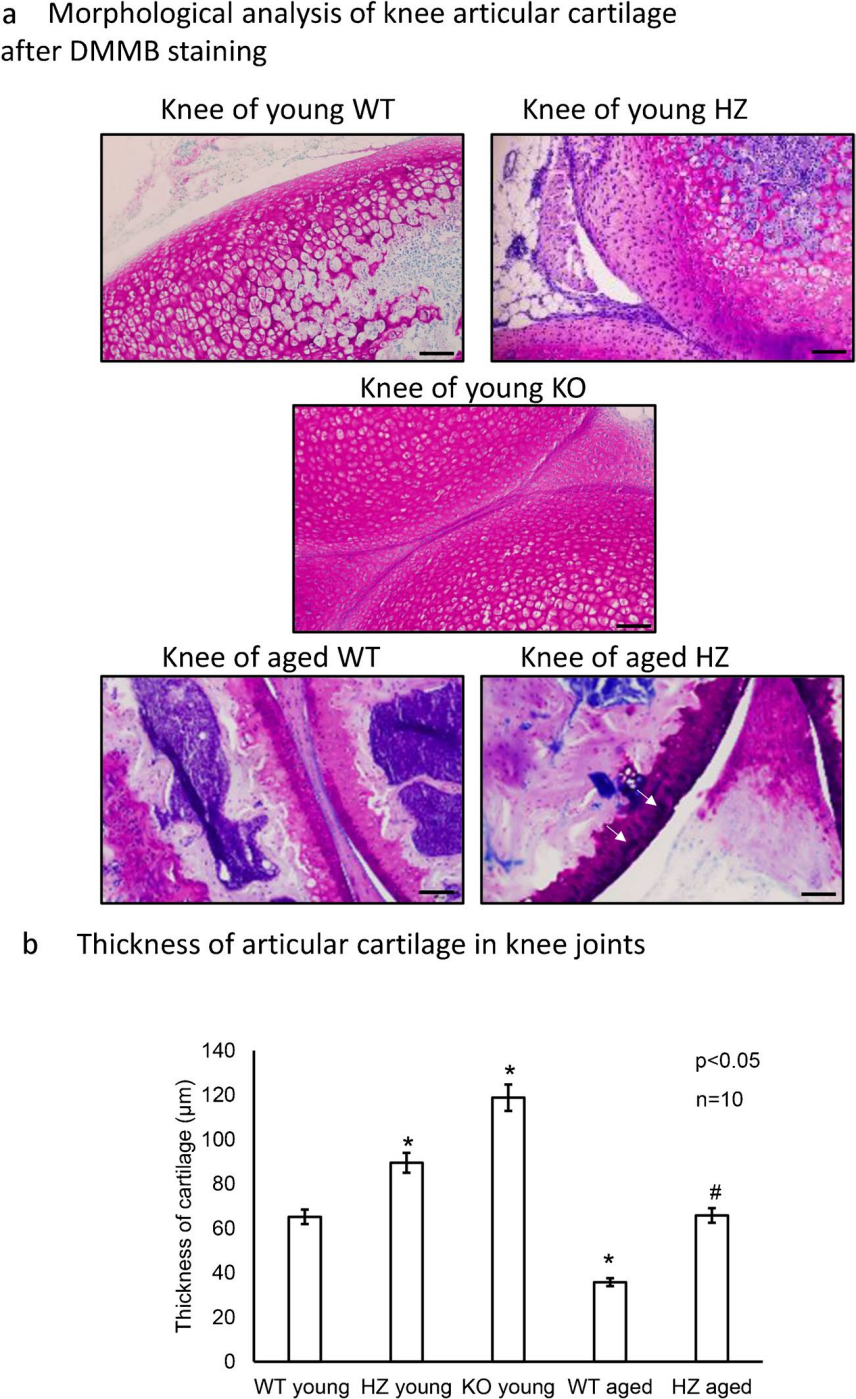
Morphological analysis of knee articular cartilage. **a** Dimethyl-methylene blue (DMMB) staining was used to visualize glycosaminoglycan expression differences. Following groups were set: knee of young WT, knee of young HZ, knee of young KO, knee of aged WT, and knee of aged HZ. Original magnification was 10 ×. Scale bar: 500 µm. Representative data of at least 5 independent experiments. **b** Thickness of articular cartilage and measurement of metachromatic area with DMMB staining. Number of articular cartilage shown at least *n* = 5 cases. Asterisks indicate significant (*p < 0.05) difference in cartilage thickness compared to the WT young controls and # indicate significant (^#^*p* < 0.05) difference compared to the WT aged controls

For identification of metachromatically stained articular cartilage DMMB staining was performed. From the PAC1 homozygous KO mice only five knee joint samples were harvested because of the high mortality of animals. Fifteen samples of PAC1-R WT and HZ knee joint samples were used to analyze the thickness of metachromatically stained cartilage. In young WT mice the knee joints showed normal morphology, with a thick and distinct articular facet. Superficial, intermediate and deep zones started to separate from the forming growth plate. Cells in the intermediate zone were forming groups and started to be organized into columns in the deep zone. The extracellular matrix showed intensive metachromasia, without any morphological disorder (Fig. 1a). Interestingly, in PAC1 HZ mice, the superficial zone of articular cartilage was not well distinguishable but a thick intermediate zone developed. The surface of the articular cartilage was not always physiologically arched, and some waves were visible without lesions or any other disorders. In the intermediate zone more cells appeared compared to WT mice with an altering metachromasia in the ECM. The deep zone was well identifiable with the classical columnar structure and the growth plate started to separate as it was visible in the WT mice (Fig. 1a). In PAC1 KO homozygous littermates, the cartilage surface was arched and did not show any morphological alterations. On the other hand, the superficial zone was thin and not well defined, but the intermediate zone was thicker compared to WT littermates with a large number of cells and intensive metachromasia. The deep zone of young PAC1 KO mice was barely identifiable and the growth plate just started to separate. Moreover, the hypertrophic cells were not arranged into regular columns (Fig. 1a).

In aged mice the metachromatically stained articular cartilage became thinner and the intensity of the staining was variable within the tissue. The superficial layer was extremely thin, sometimes barely detectable in WT animals. Therefore, the surface integrity of the articular cartilage was weaker and the arch of the surface became a little wavy. The intermediate zone was visible with a smaller number of cells and occasionally paler metachromasia and the deep zone was very thin with a few numbers of hypertrophic cells in WT mice (Fig. 1a). Interestingly, in the aged PAC1 HZ animals the superficial zone showed a characteristic and strong metachromasia. Moreover, the intermediate zone of knee joint’s articular cartilage lost its solid metachromatic color and stronger plaque-like structures appeared with darker staining. The deep zone contained hypertrophic cells, but the columnar orientation was barely visible. As the PAC1 homozygous animals died after a few weeks of life, no articular cartilage was investigated (Fig. 1a).

To provide semiquantitative data about the cartilage integrity, the thickness of metachromatically stained cartilage was measured with a simple mathematic formula published earlier (Szegeczki et al. 2019). In young animals, the cartilage thickness was measured till the separation of the growth plate. Interestingly, the thickness of the metachromatic area was elevated in HZ mice, and a further significant thickening was detected in PAC1-R KO mice compared to young WT animals (Fig. 1b). The aged knee joint’s cartilage was definitely thinner in aged WT individuals compared to the young WTs. On the other hand, a significant thickness increase was detected in aged HZ animals compared to aged WT littermates (Fig. 1b).

### Morphological analysis of articular cartilages of the foot

**Fig. 2:**
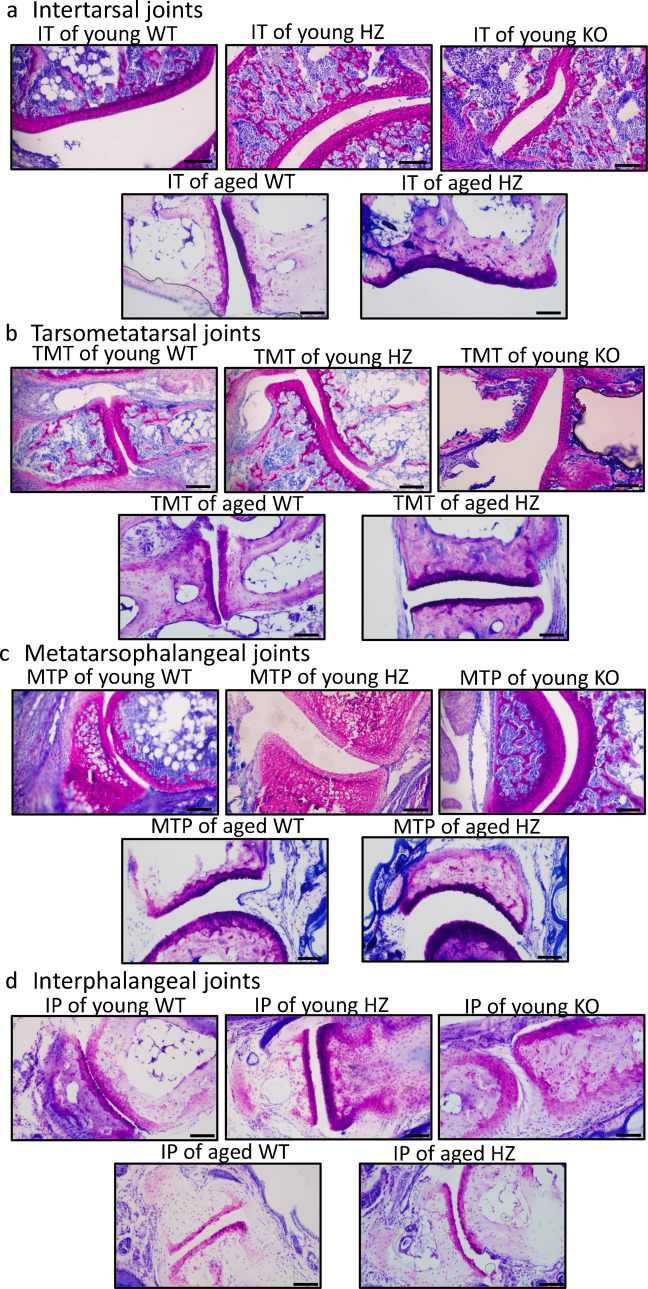
Morphological analysis of foot joints after dimethyl-methylene blue (DMMB) staining. **a** intertarsal (IT) joints, **b** tarsometatarsal (TMT) joints, **c** metatarsophalangeal (MTP) joints, and **d** interphalangeal (IP) joints. The groups were the following in every foot joint: young WT, young HZ, young KO, aged WT, and aged HZ. Original magnification was 10 ×. Scale bar: 500 µm. Representative data of at least 5 independent experiments

**Fig. 3:**
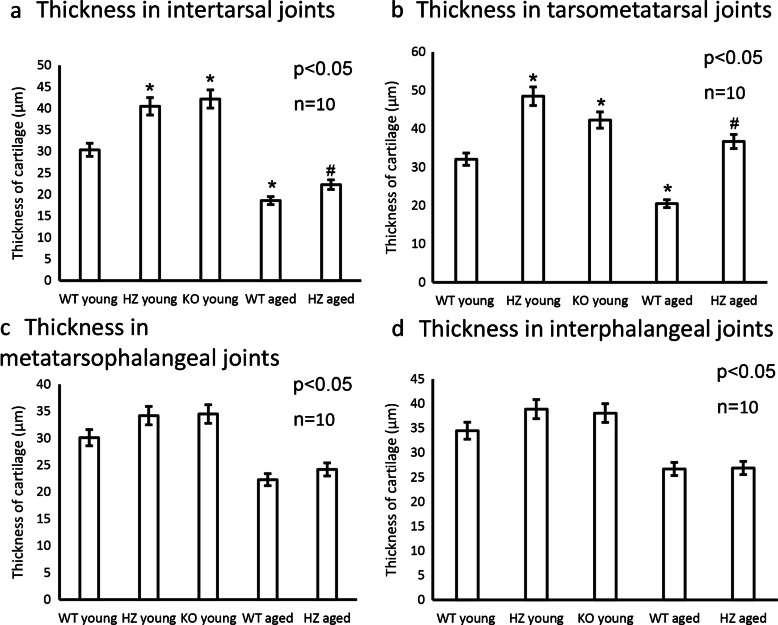
Thickness of articular cartilage, measurement of metachromatic area with DMMB staining. **a** intertarsal (IT) joints, **b** tarsometatarsal (TMT) joints, **c** metatarsophalangeal (MTP) joints, and **d** interphalangeal (IP) joints. The groups were the following in every foot joint: young WT, young HZ, young KO, aged WT, and aged HZ. Number of articular cartilage shown at least *n* = 5 cases. Asterisks indicate significant (*p < 0.05) difference in cartilage thickness compared to the WT young controls, and # indicates significant (^#^*p* < 0.05) difference compared to the WT aged controls

To prove the general function of PAC1 receptor in articular cartilage further joints were investigated, such as intertarsal (IT), tarsometatarsal (TMT), metatarsophalangeal (MTP), and interphalangeal (IP) joints. These joints can be the target of many arthritis but tend to have less dramatic radiographic manifestations. The intertarsal joints function to absorb energy in the early stance phase and carry the weight of the body in the beginning of the gait circle (Nuber 1988). Articular cartilage is physiologically thinner in intertarsal (IT) joints than in knee joints, but are also affected by the same disorders such as osteoarthritis. The IT joints showed an intensive metachromasia in young WT animals, with a well visible proliferating chondrocyte population getting to be organized into chondrons (Fig. 2a). A few hypertrophic cells were visible with dark metachromatic staining in the deep zone of young WT animals as well as in the osteoids (Fig. 2a). In PAC1 HZ mice, the metachromatic color of IT joints was lighter with large number of proliferating cells. In the deep zone some hypertrophic cells were visible and metachromatic staining was identifiable in subchondral bone and osteoids. Similar phenomena could be detected in intertarsal cartilage of young homozygous PAC1-R KO mice (Fig. 2a). The thickness of metachromatic cartilage significantly elevated in HZ and PAC1-R KO mice (Fig. 3a). In aged WT mice the metachromasia became lighter in the joint and a decreased number of chondrocytes was detected compared to young WT control (Fig. 2a). On the contrary, in aged HZ PAC1 receptor deficient mice the cartilage of intertarsal joints presented darker metachromasia and the area of metachromatic cartilage was slightly increased without reaching significant level in aged HZ mice (Figs. 2a and 3a).

Then, the tarsometatarsal (TMT) joints were investigated. These joints are relatively rigid, except for the first, which allows moderate amounts of dorsiflexion and plantar flexion. The tarsometatarsal joints carry the bodyweight during the stance phase of gait (Nuber 1988). In young WT animals these joints were relatively thin and superficial zone’s metachromasia was equally distributed as well as in the deep zone (Fig. 2b). The PAC1 HZ and PAC1 KO mice had lighter but solid metachromasia equally distributed in all zones and intensive metachromasia appeared in the subchondral bone (Fig. 2b). The number of chondrocytes was higher in the cartilage of young TMT joints (Fig. 2b). Thickness of metachromatic cartilage was significantly higher in young HZ and PAC1 KO mice. In aged animals, the cartilage in WT was lighter stained and in HZ animals had stronger metachromasia with intensive staining (Fig. 3b). The metachromatically stained cartilage was significantly thicker in aged TMT joints (Fig. 3b).

The metatarsophalangeal (MTP) joints are located between the convex head of the metatarsals and the shallow concavity of the proximal phalanges. These joints do not carry the weight of the body during the gait cycle (Nuber 1988). In young WT animals, intensive and dark metachromasia could be identified (Fig. 2c). In PAC1 HZ mouse MTP joints, the metachromatic color was lighter and intensive with large number of chondrocytes in PAC1 KO staining was darker with similar characteristics as in HZ animals (Fig. 2c). On the other hand, the cartilage thickness did not alter in young HZ and PAC1 KO littermates (Fig. 3c). In the aged WT animals thinner but darkly stained metachromatic area was visible, this metachromasia was increased in the aged HZ MTP joints (Fig. 2c). On the contrary, no significant alterations were detected in thickness of metachromatically stained cartilage (Fig. 3c).

The interphalangeal joint (IP) of the toes is synovial joint mostly works during flexion and extension of fingers without carrying the weight of the body during locomotion (Nuber 1988). Without making any differences of proximal and distal IP joints the articular cartilage was investigated. In young animals thick metachromatic cartilage was visible in WT, HZ and PACAP KO mice. A large number of chondrocytes were identifiable with a well-defined intermediate zone (Fig. 2d). No significant differences were identified in cartilage thickness between WT, HZ, and PAC1 KO littermates (Fig. 3d). In aged animals, the cartilage metachromasia was lighter (Fig. 2d), but the thickness did not alter significantly (Fig. 3d).

### Orientation of collagen was preserved in aged PAC1 HZ knee joints

**Fig. 4:**
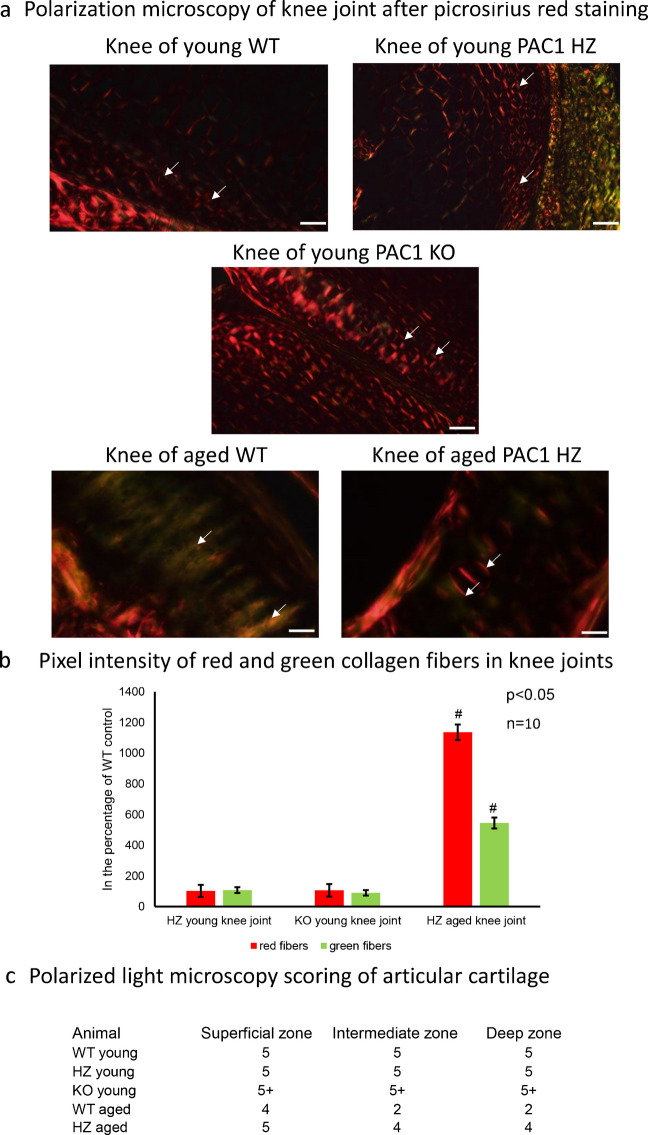
Polarization light microscopical (PLM) analysis of knee joint articular cartilage. **a** Picrosirius red staining was used to visualize the birefringence of collagen fibrils in polarized light The groups were the following: knee of young WT, knee of young HZ, knee of young KO, knee of aged WT, and knee of aged HZ. Arrows represent the maltan crosses around chondrons. Original magnification was 20 ×. Scale bar: 200 µm. Representative data of at least 5 independent experiments. **b** Red and green pixel intensity was measured in knee joints. Measurable number of articular cartilage shown at least *n* = 5 cases. Asterisks indicate significant (*p < 0.05) difference in cartilage thickness compared to the WT young respective controls, and # indicates significant (^#^*p* < 0.05) difference compared to the WT aged controls. **c** PLM scoring according to the international standards. Score of PLM represents disorientation of collagen fibers in the superficial, intermediate, and deep zones

In healthy knee joint articular cartilage, the orientation of collagen type II in the superficial zone is parallel to the surface and then it is perpendicular to it in the intermediate zone. With picrosirius red staining, collagens can be visualized without any specificity. On the other hand, with picrosirius red staining, collagen orientation can be demonstrated in red and green colors using polarization microscopy, with the polarized light plane turned with λ/4. The shiny red color represents the thick collagen fibers and thinner fibers have a light green characteristic. Around the chondrons, maltan crosses appear in polarized light indicating the physiological orientation of collagen type IV. On the surface of articular cartilage collagen exhibits a thin, uniform layer of birefringence due to parallel alignment of collagen in the superficial zone. The random arrangement of collagen II fibrils in the intermediate zone leads to a decreased birefringence with circular orientation around chondrons (Changoor et al. 2011). The presence of orthogonally arranged thin collagen II fibrils in the deep zone results in low birefringence. The measurement of thick and thin collagen fibers ratio can be a semiquantitative information about the structural integrity of collagens in the articular cartilage as well as the maltan crosses can be a good qualitative characteristic of proper cartilage ECM orientation. In WT young mice, the superficial zone in knee joints was very thin and had a pale appearance. Maltan crosses in the intermediate zone showed a physiological characteristic with an equal ratio of thick and thin collagen fibers (Fig. 4a). On the contrary, the superficial zone in knee joints of PAC1 HZ and KO mice had a well-defined sharp light appearance (Fig. 4a). The thicker collagen fibers were elevated compared to the WT young littermates (Fig. 4b) which was also supported by higher PLM score 5 (Fig. 4c). On the other hand, around the chondrons the maltan crosses were stronger and thicker than in WT individuals (Fig. 4a) which showed a bit abnormal PLM score (Fig. 4c). In aged WT mice, the superficial zone of knee joint’s articular cartilage was well defined (Fig. 4a) and had more thick red fibers running parallel with the surface compared to the young WT animals (Fig. 4b). In the intermediate zone the maltan crosses were diminished or showed abnormal characteristics in aged WT animals (Fig. 4a). Altogether, the thinner collagen fibers increased in the articular cartilage of knee joints (Fig. 4b) and PLM score was reduced to 2 (Fig. 4c). Interestingly, in aged PAC1 HZ mice, the superficial zone was more prominent and thicker with higher amount of thick fibers (Fig. 4b). The maltan crosses in the intermediate zone were a little deformed but showed relatively normal characteristics (Fig. 4a). The PLM score was 4 of aged PAC1 HZ mice, similar to young WT individuals (Fig. 4c).

### Collagen structural appearance altered in PAC1 HZ foot joints

**Fig. 5:**
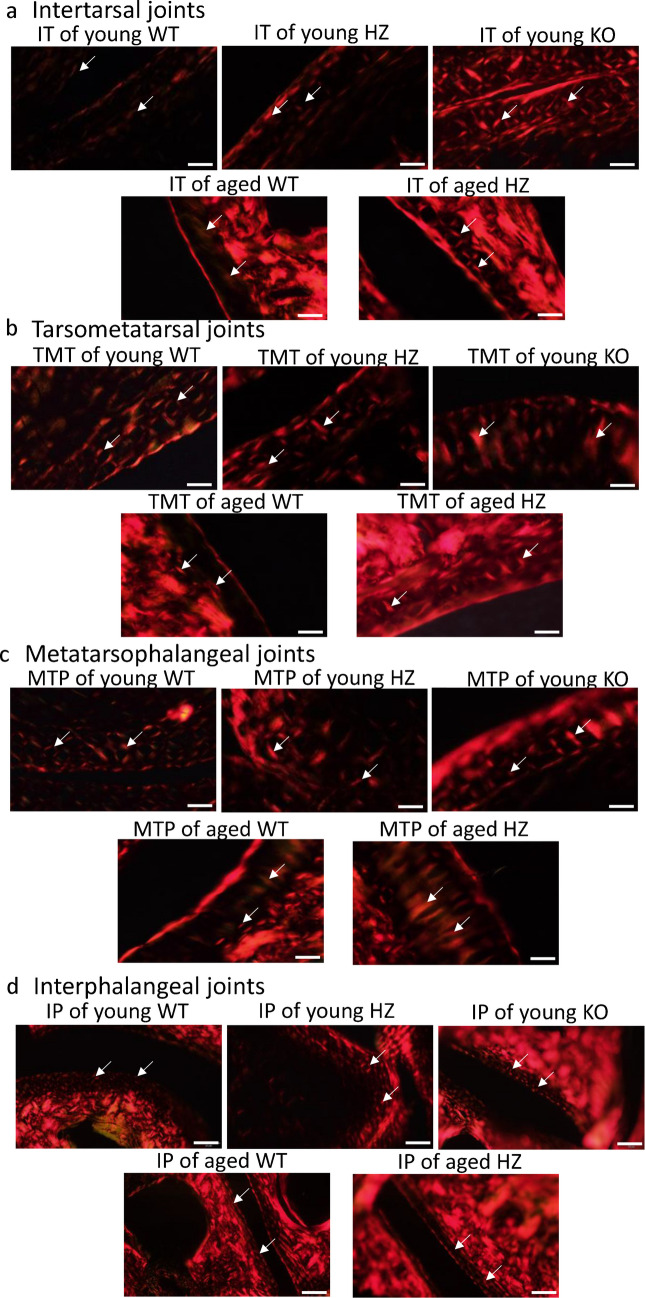
Polarization light microscopical (PLM) analysis of foot joints’ articular cartilage. **a** Picrosirius red staining was used to visualize the birefringence of collagen fibrils in polarized light. **a** Intertarsal (IT) joints, **b** tarsometatarsal (TMT) joints, **c** metatarsophalangeal (MTP) joints, and **d** interphalangeal (IP) joints. The groups were the following in every foot joint: WT, young HZ, young KO, aged WT, and aged HZ. Arrows represent the maltan crosses around chondrons. Original magnification was 20 ×. Scale bar: 200 µm. Representative data of at least 5 independent experiments

**Fig. 6:**
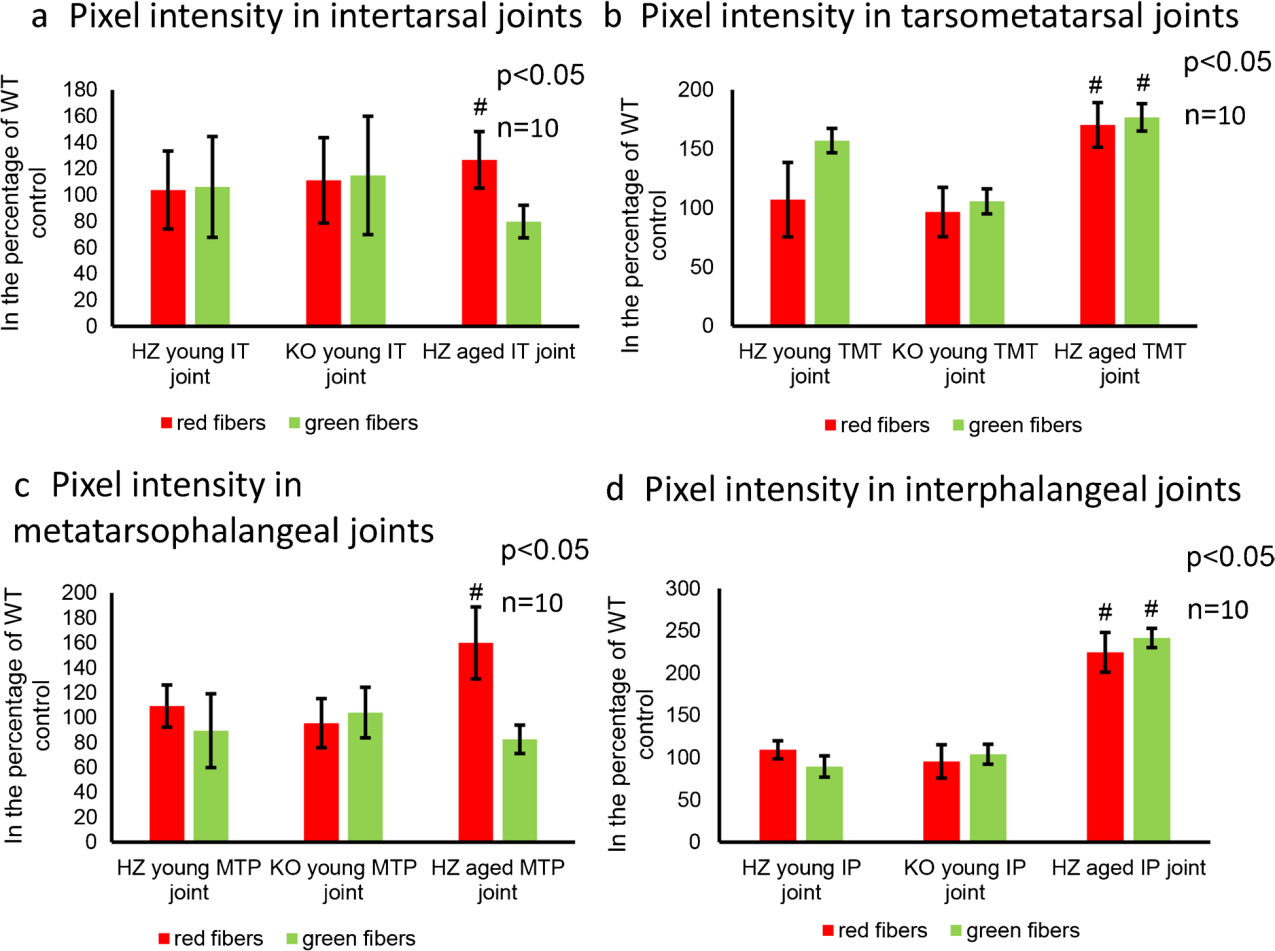
Pixel intensity of red and green collagen fibers in foot joints. **a** Intertarsal (IT) joints, **b** tarsometatarsal (TMT) joints, **c** metatarsophalangeal (MTP) joints and **d** interphalangeal (IP) joints The groups were the following in every foot joint: young WT, young HZ, young KO, aged WT and aged HZ. Measurable number of articular cartilage shown at least *n* = 5 cases. Asterisks indicate significant (*p < 0.05) difference in cartilage thickness compared to the WT young controls, and # indicates significant (^#^*p* < 0.05) difference compared to the WT aged controls

The IT joints’ superficial zone in young WT was similar to knee joints with a thin red line-like appearance (Figs. 5a and 6a). Maltan crosses were pale and regular as it was demonstrated in knee joints of young WT mice (Fig. 5a). In young PAC1 HZ the superficial zone was tendentiously thicker, and the ratio of thicker collagen fibers was significantly higher (Figs. 5a and 6a). The maltan crosses were more definite compared to young WT individuals (Fig. 5a). Interestingly, the superficial zone of articular cartilage was the strongest in young PAC1 KO mice as well as the maltan crosses had thick and prominent characteristics (Fig. 5a). The amount of thick collagen fibers was significantly higher than in WT littermates (Fig. 6a). In aged WT mice the superficial zone of IT joints was thicker than in young WT animals but thinner than in young PAC1 KO mice (Fig. 5a). Thick collagen fibers were less abundant in the intermediate zone and maltan crosses were almost unidentifiable in the WT mice (Fig. 6a). On the contrary, in PAC1 HZ animals, the superficial zone of articular cartilage was significantly thicker (Fig. 5a) and the ratio of red collagen fibers was higher than in aged WT mice (Fig. 6a). Moreover, regular maltan crosses were detected in the intermediate zone in aged PAC1 HZ IT joints (Fig. 5a).

The superficial zone of TMT joints was thin and well distinguishable; moreover, well-characterized maltan crosses were detected in the intermediate zone in young wild type animals (Fig. 5b). The ratio of thick collagen fibers was significantly higher in this joint (Fig. 6b). Interestingly, young PAC1 HZ and KO animals did not show alterations in the thickness and orientation of collagens in the superficial zone (Fig. 6b). The maltan crosses were also well detectable in the intermediate zone of PAC1 HZ individuals, but some abnormal elongated deformation was identified in the PAC1 KO animals (Fig. 5b). In aged WT individuals, a thin superficial zone was detected with the reduction of birefringence as well as a decreased amount of physiological maltan crosses (Fig. 5b). The number of thick collagen fibers was lower compared to young WT animals (Fig. 6b). On the contrary, in aged PAC1 HZ mice, the superficial zone was significantly thicker, and stronger birefringence was visible between the chondrons, while the maltan crosses were well defined and regularly shaped (Fig. 5b).

The MTP joints of young WT animals the superficial zone was almost undetectable and well characterized maltan crosses appeared in the intermediate zone (Fig. 5c). In young PAC1 HZ and KO animals a thick layer of strong birefringence was identified (Fig. 5c). Interestingly, the ratio of thick collagen fibers did not increase significantly compared to WT animals (Fig. 6c). The maltan crosses of the intermediate zone showed regularity as it was also detected in the WT mice (Fig. 5c). In aged WT individuals the superficial zone was well detectable as well as in aged PAC1 HZ mice (Fig. 5c). On the other hand, both aged WT and HZ littermates the maltan crosses showed deformities and the amount of green, thinner collagens elevated in the intermediate zone (Figs. 5c and 6c).

The superficial zone articular cartilage in IP joints of young WT animals was similar to the MTP joints (Fig. 5d). On the other hand, the intensive thickness alteration of superficial zone was not detectable in young PAC1 HZ and KO mice (Fig. 5d). The thickness of collagen fibers did not alter in these animals compared to the young WT individuals (Fig. 6d). In aged WT and HZ mice, the articular cartilage had a thin superficial zone with an altered maltan cross architecture in the intermediate zone (Fig. 5d). Furthermore, the amount of thin collagen fiber increased in the aged IP joints (Fig. 6d).

### Nuclear localization of P-Sox9 in aged articular cartilage

**Fig. 7:**
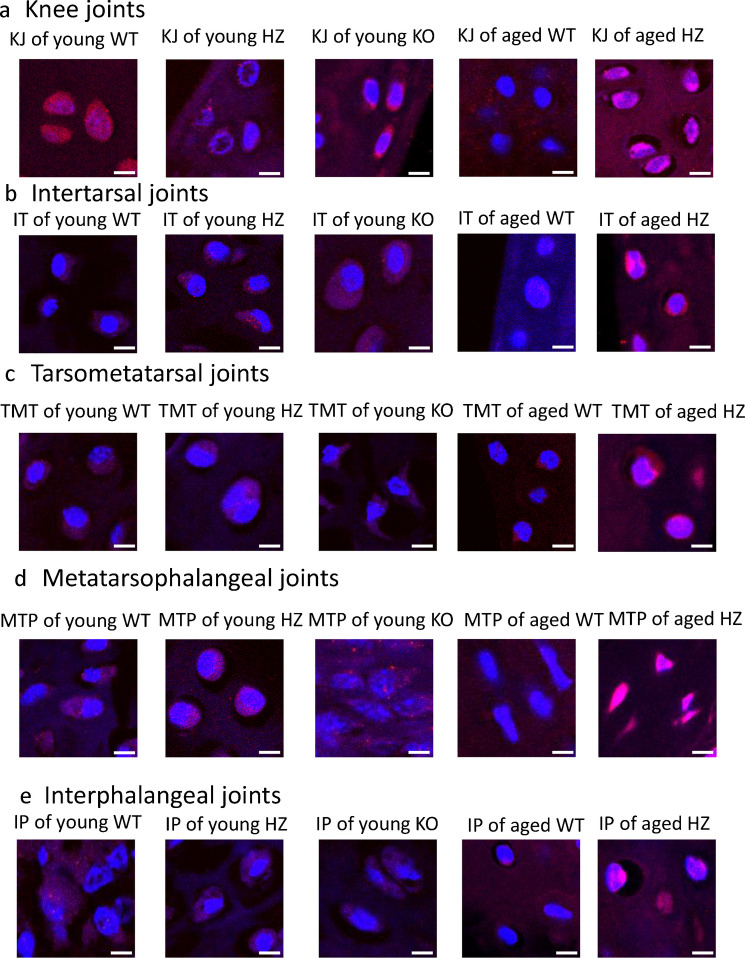
P-Sox9 immunohistochemistry. **a** Knee joint (KJ), **b** intertarsal (IT) joints, **c** tarsometatarsal (TMT) joints, **d** metatarsophalangeal (MTP) joints, and **e** interphalangeal (IP) joints. The groups were the following in every foot joint: young WT, young HZ, young KO, aged WT, and aged HZ. Original magnification was 60 ×. Scale bar: 5 µm. Representative data of 3 independent experiments

Activation of PAC1 receptor can induce Sox9 phosphorylation, which is translocated into the nuclei of chondrocytes to trigger the expression of ECM components. Therefore, the nuclear translocation of phosphorylated Sox9 was followed by confocal microscopy. In young individuals, the translocation was consistently detected with no differences found in any of the joints (Fig. 7a–e). Strong signals were present in the cytoplasm and in the nuclei of chondrocytes. Independently from the location of the joint, the phosphorylated form of Sox9 was translocated equally to the nucleus without showing alterations in young PAC1 HZ and KO animals (Fig. 7a–e). After the end of bone elongation and development, the chondrocytes decrease the expression of Sox9. Subsequently, the phosphorylated form of the transcription factor also decreases, as it was detected in aged WT animals (Fig. 7a–e). Although the expression of P-Sox9 was detected in the cytoplasm of chondrocytes in knee (Fig. 7a), IT, TMT, MTP, and IP joints, its nuclear translocation was barely detectable in young animals (Fig. 7b–e). In aged PAC1 HZ animals, independently from the joint location, elevated cytoplasmic presence of P-Sox9 was detected (Fig. 7a–e). The nuclear localization of P-Sox9 was also increased in the chondrocytes, independently from the location of the joints. The immunopositivity of the P-Sox9 transcription factor was almost at the same level as in the young experimental groups (Fig. 7a–e).

## Discussion

The primary role of articular cartilage is to facilitate smooth movement at joints by providing a low-friction surface. This smooth articulation is critical for joint mobility and is achieved through the interaction between the cartilage surface and the synovial fluid that lubricates the joint, reducing friction during motion (Camarero-Espinosa et al. 2016). Additionally, articular cartilage acts as a shock absorber, evenly distributing the mechanical loads exerted during activities such as walking, running and jumping. This property minimizes the risk of damage to the underlying bone and surrounding tissues, preventing excessive wear and tear during repetitive movements (Krakowski et al. 2024). Despite its vital role in joint function, articular cartilage is susceptible to damage and degeneration. Conditions such as osteoarthritis, traumatic injuries and other joint disorders often lead to cartilage degradation, resulting in pain, inflammation, and reduced mobility. The avascular nature of articular cartilage poses significant challenges for healing and repair. Unlike other tissues, damaged cartilage has a limited capacity for self-repair due to the low metabolic activity of chondrocytes and the sparse blood supply (Ghosh and Cheras 2001). We have published that during aging of human knee articular cartilage, cartilage thickness declines in parallel with PAC1 receptor expression, although no direct correlation between these two phenomena has been demonstrated (Racz et al. 2025). However, contemporary OA research has increasingly moved beyond focusing solely on cartilage thickness. Current priorities emphasize overall joint health, function, and symptom management—particularly pain reduction and maintenance of mobility (Jang et al. 2021). Interventions aimed solely at restoring cartilage thickness have largely failed to yield meaningful clinical improvements (Jansen et al. 2022). Thus, while cartilage thinning remains a measurable phenomenon in OA (Racz et al. 2025), it is no longer considered the central therapeutic target or the primary focus of most research agendas.

Articular cartilage is primarily composed of extracellular matrix (ECM), which provides its unique mechanical properties. The ECM is composed of water (approximately 70–80%), collagen fibers, PGs, and non-collagenous proteins. The predominant collagen type in articular cartilage is type II, which forms a dense mesh that provides tensile strength. These collagen fibers are organized in a specific manner, with orientation varying between zones: the superficial zone, middle zone and deep zone (Bhosale and Richardson 2008). The hind limb of animals is an intricate structure designed to bear weight and facilitate movement. The weight-bearing capacity of the joints in the hind limb, including knee, ankle and foot joints, is a critical aspect of mammalian biomechanics. The alignment, range of motion and stability of the joints play significant roles in their weight-bearing ability (Wentink 1977; Helms et al. 2009; Nicola and Jewison 2012). Optimal biomechanics ensure even weight distribution across the joint surfaces, reducing the risk of injury. Insufficient weight-bearing capacity increases the risk of joint injury, muscle strain and falls. Older adults, who may experience decreased joint function and instability, are particularly vulnerable to injuries related to inadequate weight-bearing capacity (Logerstedt et al. 2022).

PACAP and its receptors, PAC1-R, VPAC1-R and VPAC2-R have been identified in hyaline cartilage and they play a pivotal role in chondrogenic differentiation (Juhasz et al. 2014a). In the central nervous system, PAC1 receptor is involved in neuronal survival, synaptic plasticity and neurotransmitter release (Shioda et al. 2006). It has been shown to protect neurons from excitotoxicity, oxidative stress and apoptosis (Shioda et al. 2006; Racz et al. 2010; Seaborn et al. 2011). Additionally, the PAC1-R is implicated in the regulation of circadian rhythms, sleep–wake cycles and stress responses (Butcher et al. 2005; Wong and Schumann 2012). Dysfunction of the PAC1 receptor has been associated with various neurological disorders, such as epilepsy, Alzheimer’s disease and schizophrenia (Rocha-Martins and Njaine 2013; Shen et al. 2013; Schaler et al. 2021). In the endocrine system, PAC1-R regulates the release of various hormones, including adrenocorticotropic hormone (ACTH), growth hormone (GH) and thyroid-stimulating hormone (TSH). Activation of the PAC1 receptor in the pituitary gland stimulates the release of these hormones, which control metabolism, growth and reproduction (Arimura 1998; Okada et al. 2007). Abnormalities in the PAC1 receptor signaling have been linked to hormonal imbalances, pituitary adenomas and other endocrine disorders. The PAC1 receptor is an important therapeutic target for the treatment of various diseases and disorders. Modulation of the PAC1 receptor signaling pathways has the potential to alleviate symptoms and improve outcomes in neurological, endocrine and immune disorders (Moody et al. 2016). Pharmacological agents that target the PAC1 receptor have been developed and tested in a preclinical study in migraine (Tajti et al. 2014).

However, it has been published that PACAP serves as a positive regulator of in vitro chondrogenesis through the canonical signaling pathway that culminates in the activation of the Sox9 transcription factor, along with others such as CREB or SHH signaling (Juhasz et al. 2014a, 2015b; Szegeczki et al. 2019). Sox9 is essential for chondrogenesis as it promotes the expression of aggrecan and collagen type II. Additionally, the normal expression and functioning of Sox9 postnatally are vital for maintaining the integrity of the cartilage matrix, which can help to prevent osteoarthritis (OA) and keep the growth plates open through continuous expression (Song and Park 2020). The disorders of Sox9 function can lead the different musculoskeletal diseases, and its lack can lead to bone and cartilage deformation (Lefebvre et al. 2019). Despite its critical role in matrix production, the expression of this transcription factor during aging has not yet been investigated. As the transcription factor can be activated by PAC1 receptor it can be a question of interest whether its phosphorylation via PACAP signaling is crucial in chondrogenesis or it has a multifactorial activation.

Moreover, it is also known that PACAP signaling cascade and its crosstalks have a balancing function in various processes. The chondroprotective effects of PACAP are well-documented and PACAP is essential for proper cartilage formation (Szegeczki et al. 2019). The PAC1 receptor has been identified in chondroprogenitor cells and it has been followed in aged animals, suggesting that PACAP may promote extracellular matrix synthesis, highlighting its positive role in cartilage development (Szegeczki et al. 2019). Interestingly, in this study, complete or partial absence of PAC1-R resulted in cartilage thickening and elevated matrix production in young and aged animals. On the other hand, the P-Sox9 nuclear presence increased in aged PAC1 deficient animals. These findings suggest the balancing effect of PACAP signaling in chondrogenic differentiation (Szentleleky et al. 2019). It has been published that the absence of PACAP neuropeptide provokes the earlier formation of OA and elevation of PAC1-R expression (Szegeczki et al. 2019). It refers to the fact that chondrocytes compensate for the lack of the neuropeptide to rebalance the physiological conditions with activation of other crosstalk mechanisms (Szegeczki et al. 2025). This balancing effect has also been shown in the dynamic matrix formation and degradation as PACAP can regulate MMP activation to induce cartilage specific matrix formation (Szentleleky et al. 2019). In high density chondrogenic cultures, the inhibition of PACAP receptors by PACAP 6–38 resulted in an increase of matrix production, which also suggests that the imbalance of PACAP signaling induces an activation of signaling crosstalk mechanisms which may overactivate proper matrix production (Juhasz et al. 2014a). Consequently, these findings also suggest that PAC1-R inactivation during in vitro cartilage formation may trigger alternative signaling pathways that compensate for reduced PAC1-R function. We have additionally published that addition of PACAP 1‑38 in cell culture induces increased expression of VPAC receptors (Juhasz et al. 2014a), further supporting the notion that the neuropeptide can activate VPAC-mediated pathways and modulate the balancing signaling of PACAP during chondrogenesis. This can also give an explanation for the contradiction that the inhibition of PAC1 receptor by PACAP 6–38 in certain conditions leads to an agonistic behaviour such as in cytotrophoblast cells (Reglodi et al. 2008). It is known about PACAP release that it is involved in the regulation of stress hormones, such as cortisol, and can help to modulate stress responses. These effects also refer to the shift in the steady state of PACAP balance, which induces the overactivation of survival signaling, and, through this mechanism, can induce antiapoptotic effects (Fabian et al. 2012). PACAP in vivo concentration is in the picomolar range also suggesting a short and well-balanced activation of the receptors (Matsuda et al. 2003). Furthermore, the activation of the PAC1 receptor is not the only way of signaling cascade induction as VPAC1 and VPAC2 receptors can also bind PACAP. The binding affinity of the PAC1 receptor is higher than that of VPAC1 and VPAC2, but these two receptors can bind both VIP and PACAP (Doan et al. 2011). As VPAC1 and VPAC2 receptor expression has been demonstrated in hyaline cartilage (Juhasz et al. 2014a), it is likely that PAC1 receptor can be substituted by VPAC1 and VPAC2 receptors to keep the balance of signalization. As VPAC1-R activation also elevates the activity of PKA, it is not a surprise that Sox9 phosphorylation can be elevated in the absence of PAC1-R (Gomariz et al. 2019). Through various signaling pathways, VPAC1 plays roles in different signaling pathways via which it can regulate vasodilation, modulation of immune responses, circadian rhythms, gastrointestinal motility and neuroprotection, implying its possible function in substitution of PAC1-R (Fung et al. 2014; Russo et al. 2015; Gomariz et al. 2019; Ivic et al. 2019). Although it is notable that only aged PAC1 HZ mice were investigated, which indicates a partial disturbance of PACAP signalization resulting in a set of imbalanced effects. When PAC1 signaling is reduced, PACAP may preferentially activate VPAC receptors, maintaining or even enhancing PKA activity. This could explain why nuclear P-Sox9 localization was elevated rather than diminished. Our previous results emphasize a balancing role of PACAP signaling, indicating that PAC1-R finely regulates matrix production and degradation, or controls MMP activity (Szentleleky et al. 2019). Without proper PAC1 signaling, some crosstalk pathways may become overactivated, potentially increasing matrix production. Notably, PAC1-R can also couple to Gq proteins, which induce phospholipase C (PLC) activation. Through IP_3_, this pathway increases CaMK activation (Zhang et al. 2022; Jansen et al. 2025), which can phosphorylate Sox9. Additionally, MAPK pathways, such as ERK signaling (Journot et al. 1998), can stabilize or activate Sox9 transcription, and imbalances in these pathways can further contribute to Sox9 activation (Yoshida et al. 2020). Moreover, mechanical loading activates integrins, focal adhesion kinase (FAK), ERK, and p38 MAPK pathways, which can enhance Sox9 transcriptional activity or stability (Hirose et al. 2020). If PAC1 normally modulates load-induced signaling, its disruption may shift the balance toward enhanced mechanosensitive activation of Sox9.

Based on these observations, it can be suggested that the absence of one receptor of the complex surfaceome does not definitely alter signalization in a negative way. Although it has been published that PAC1 deficiency reduces chondrogenesis in atherosclerotic plaques in hypercholesterolemia (Blumm et al. 2023). It also shows that the more complex distraction of a balanced system leads to a stronger manifestation (Blumm et al. 2023). In our results it is also visible that in joints playing an important role in weight-bearing capacity had significant alterations, while the mechanically not affected joints did not show significant cartilage structure disorders. Indeed, mechanical load has been demonstrated to have positive effects on chondrogenesis (Juhasz et al. 2014c) and without physiological physical activity abnormal cartilage formation can occur (Felsenthal and Zelzer 2017). On the other hand, PACAP has been demonstrated to prevent the harmful effect of mechanical overload and to decrease the activity of matrix degrading enzymes during mechanical stress in vitro (Juhasz et al. 2015b; Szentleleky et al. 2019). It has been also detected that the expression of PAC1-R increased during mechanical load of chondrogenic cell cultures (Juhasz et al. 2015b). This also supports that PACAP can play an important role in mechanotransduction through regulation of mechanical force-induced matrix production. Balancing effect of PACAP via PAC1-R binding in mechanical stress is to shift the collagen production into a hyaline cartilage-specific way with the inhibition of collagen production specific for hypertrophic or calcification zone (Juhasz et al. 2015b; Lauretta et al. 2020). This raises a further question whether the thickened cartilage in PAC1 gene deficient mice is a positive result that maintains the structure of healthy cartilage, or this thickening is the increase of the fibrous tissue resulted by the stronger mechanical effects. These facts are supported by our results as in HZ animals less metachromasia was detected and some wavy articular surface was demonstrated, also showing the disturbance of matrix integrity in the partial dysfunction of PACAP signaling pathways.

Altogether our result highlights the importance of PACAP signalization in proper articular cartilage formation and also suggests that precise timing and activation of PAC1-R is essential for physiologically strong hyalin cartilage production (Lauretta et al. 2020). The activation of PAC1-R can be a balancing pathway which can regulate the mechanical force induced matrix production and degradation. These results further support the theory of PAC1-R induced cartilage regeneration can be a target of new pharmacological therapies in cartilage disorders such as osteoarthritis (Grassel and Muschter 2018).

## Supplementary Information

Below is the link to the electronic supplementary material.

ESM1 (PNG 2.63 MB)

High Resolution Image (TIF 10.5 MB)

ESM2 (PNG 4.36 MB)

High Resolution Image (TIF 17.4 MB)

## Data Availability

No datasets were generated or analysed during the current study.

## Abbreviations

AC: Adenylate cyclase
ACTH: Adrenocorticotropic hormone
ADAMTS4: ADAM metallopeptidase with thrombospondin type 1 motif 4
BSA: Bovine serum albumin
CREB: CAMP response element-binding protein
DAPI: 4′,6-Diamidino-2-phenylindole
DMMB: Dimethyl-methylene blue
DPX: Water-free mounting medium for microscopy
ECM: Extracellular matrix
EDTA: Ethylene diamine tetra-acetic acid
GAG: Glycosaminoglycan
GH: Growth hormone
HZ: Heterozygous
IP: Interphalangeal joint
IT: Intertarsal joint
KO: Knock out
MMP: Matrix metalloproteinase
MTP: Metatarsophalangeal joint
NA: Numerical aperture
OA: Osteoarthritis
PAC1-R: Pituitary adenylate cyclase-activating polypeptide type I receptor
PACAP: Pituitary adenylate cyclase activating polypeptide
PBS: Phosphate-buffered saline
PBST: Phosphate-buffered saline supplemented with 1% Tween-20
PCR: Polymerase chain reaction
PG: Proteoglycan
PLM: Polarization light microscopy
PKA: Protein kinase A
SHH: Sonic Hedgehog
Sox: SRY-realated HMG-BOX gene
TMT: Tarsometatarsal joint
TSH: Thyroid-stimulating hormone
VIP: Vasoactive intestinal peptide
VPAC-R: Vasoactive intestinal peptide receptor
WT: Wild type
