# Supplementary figures and images for: Articular cartilage thickness alterations in hind limb of young and aged PAC1 gene-deficient mice
Source: Cell Tissue Res. 2026 Apr 20;404(1):5. doi: 10.1007/s00441-026-04066-5 (PMC13092545; doi:10.1007/s00441-026-04066-5)

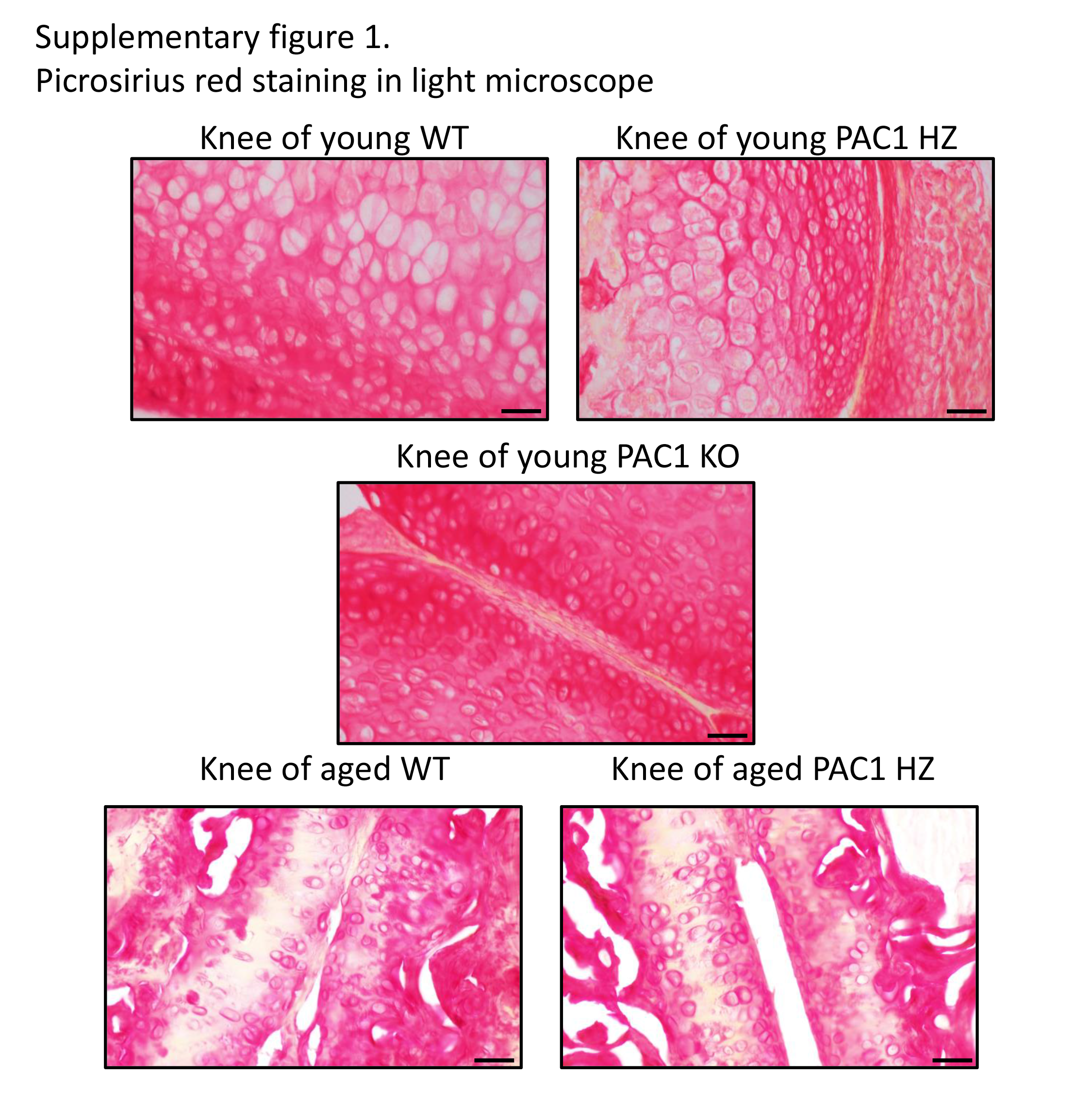

Supplement: Supplementary file 1 — (PNG 2.63 MB) [file 441_2026_4066_Fig8_ESM.png]

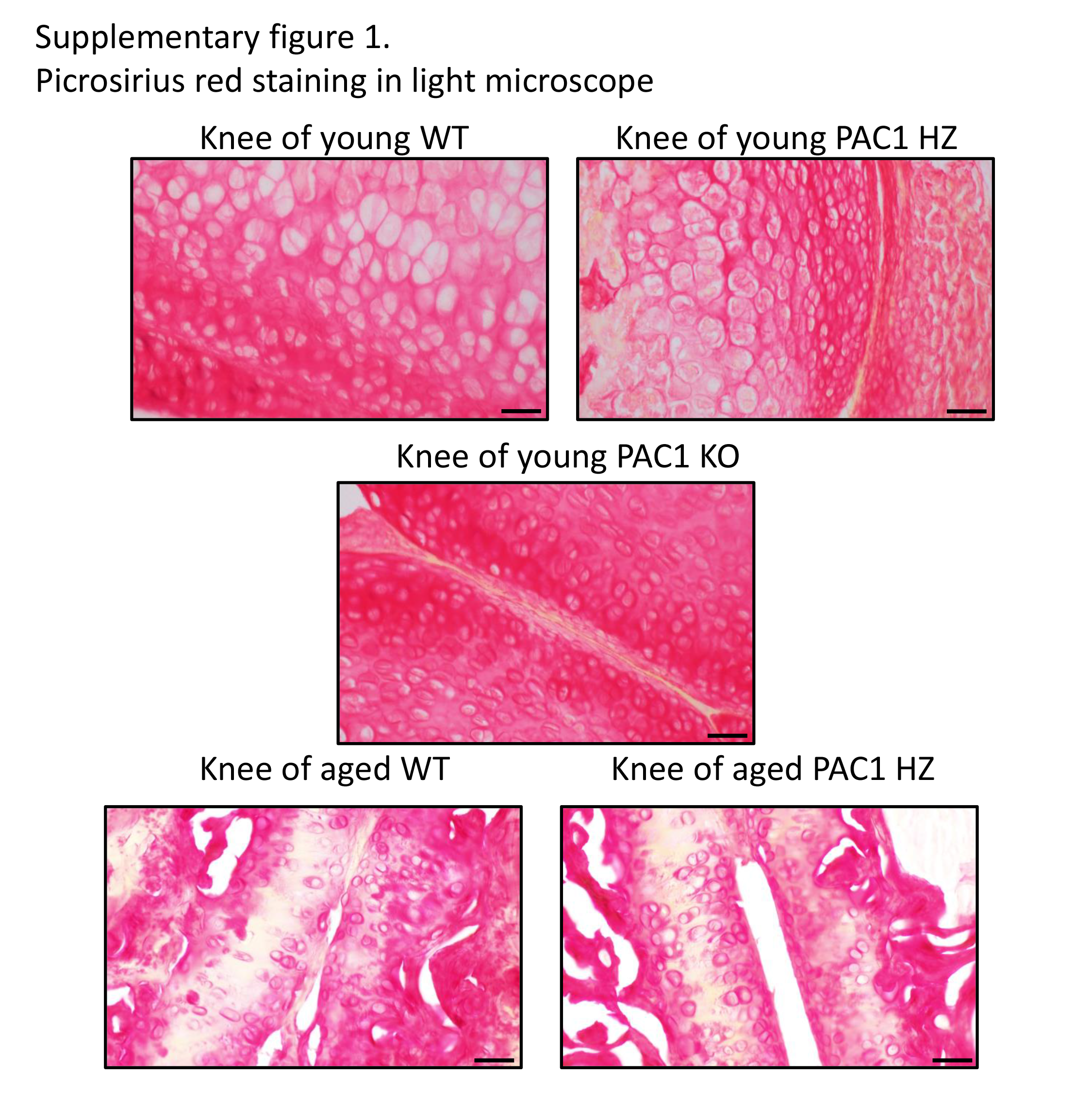

Supplement: Supplementary file 2 — High Resolution Image (TIF 10.5 MB) [file 441_2026_4066_MOESM1_ESM.tif]

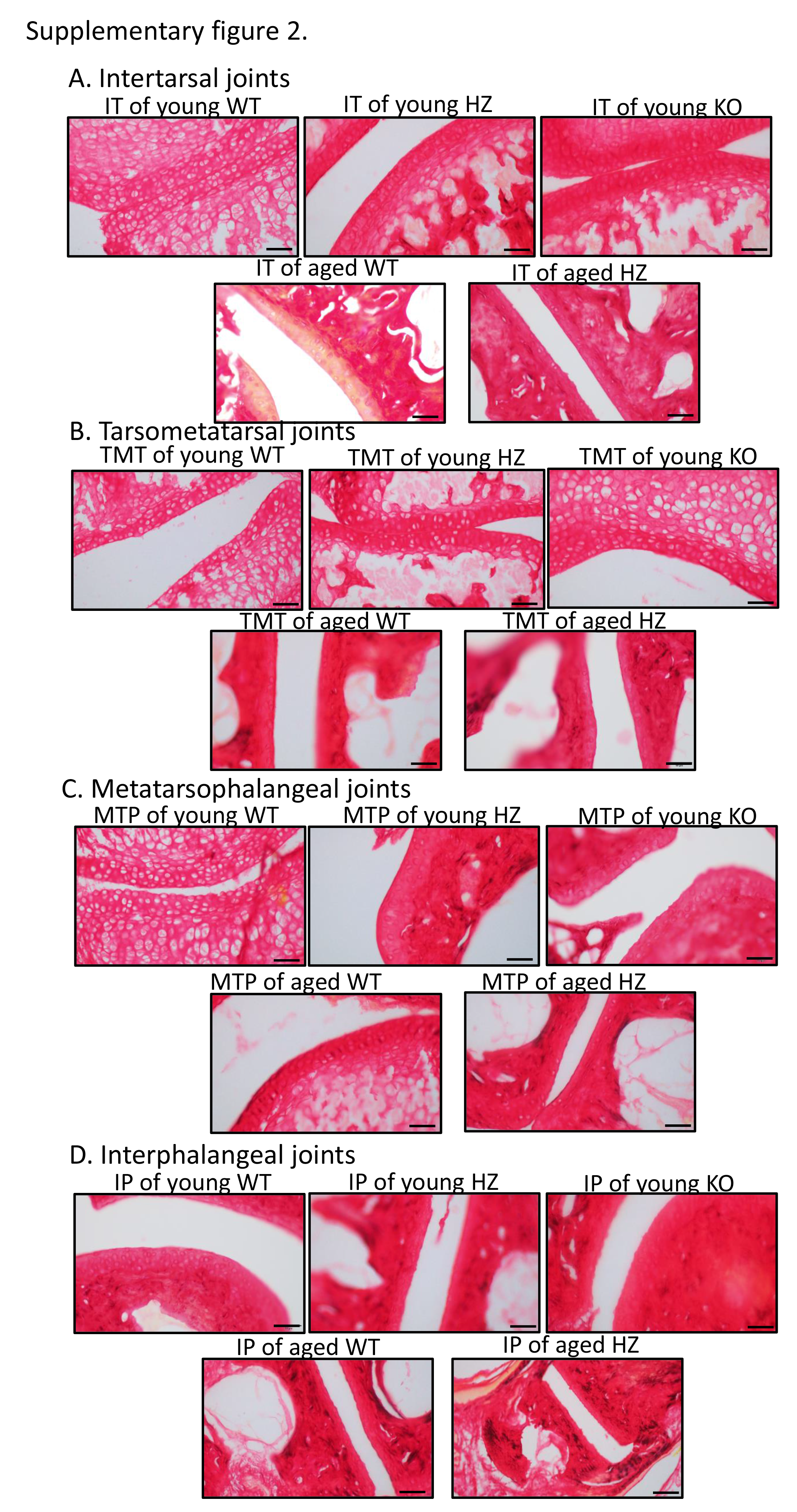

Supplement: Supplementary file 3 — (PNG 4.36 MB) [file 441_2026_4066_Fig9_ESM.png]

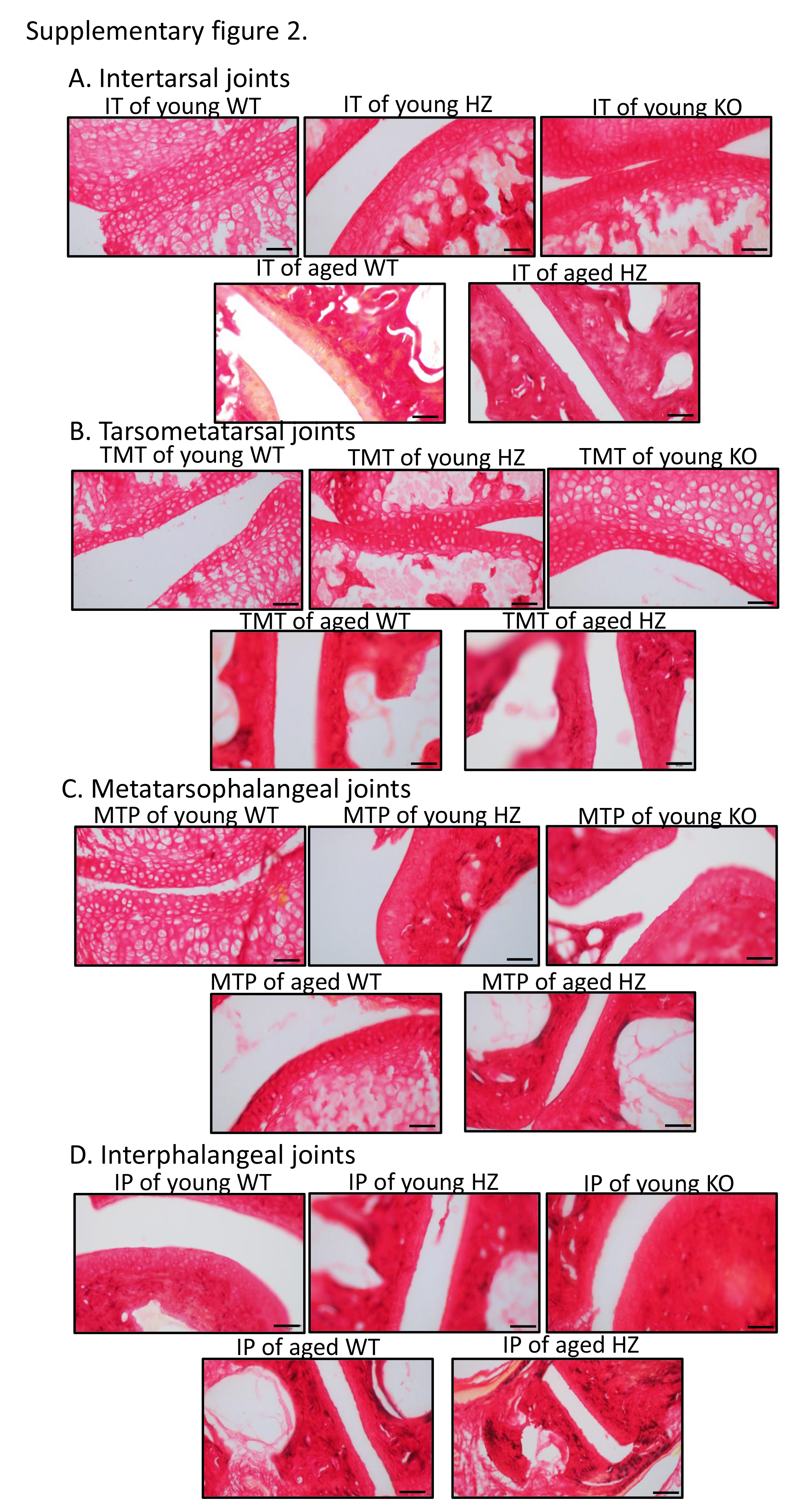

Supplement: Supplementary file 4 — High Resolution Image (TIF 17.4 MB) [file 441_2026_4066_MOESM2_ESM.tif]
